# Chemical Diversity and Biological Activities of Marine Sponges of the Genus *Suberea*: A Systematic Review

**DOI:** 10.3390/md17020115

**Published:** 2019-02-12

**Authors:** Amr El-Demerdash, Atanas G. Atanasov, Olaf K. Horbanczuk, Mohamed A. Tammam, Mamdouh Abdel-Mogib, John N. A. Hooper, Nazim Sekeroglu, Ali Al-Mourabit, Anake Kijjoa

**Affiliations:** 1 Institut de Chimie des Substances Naturelles, CNRS UPR 2301, Univ. Paris-Sud, University of Paris-Saclay, 1, Avenue de la Terrasse, 91198 Gif-Sur-Yvette, France; Ali.ALMOURABIT@cnrs.fr; 2 Organic Chemistry Division, Chemistry Department, Faculty of Science, Mansoura University, 35516 Mansoura, Egypt; mmdhbdlmgb@gmail.com; 3 Department of Pharmacognosy, University of Vienna, 1090 Vienna, Austria; atanas.atanasov@univie.ac.at; 4 Institute of Genetics and Animal Breeding of the Polish Academy of Sciences, 05-552 Jastrzebiec, Poland; 5 Faculty of Human Nutrition and Consumer Sciences, Warsaw University of Life Sciences, 02-776 Warsaw, Poland; ollafson@onet.pl; 6 Department of Pharmacognosy and chemistry of natural products, Faculty of Pharmacy, National and kapodistrian University of Athens, Panepistimiopolis Zografou, 15771 Athens, Greece; mat01@fayoum.edu.eg; 7 Department of Biochemistry, Faculty of Agriculture, Fayoum University, 63514 Fayoum, Egypt; 8 Queensland Museum, P.O. Box 3300, South Brisbane BC, QLD 4101, Australia; john.hooper@qm.qld.gov.au; 9 Department of Food Engineering, Faculty of Engineering and Architecture, Killis 7 Aralik University, 79000 Kilis, Turkey; nsekeroglu@gmail.com; 10 ICBAS—Instituto de Ciências Biomédicas Abel Salazar & CIIMAR, Universidade do Porto, Rua de Jorge Viterbo Ferreira, 228, 4050-313 Porto, Portugal

**Keywords:** marine sponges, Verongida, *Suberea*, bromotyrosine derivatives, bioactivities, biosynthesis

## Abstract

Marine natural products (MNPs) continue to be in the spotlight in the global drug discovery endeavor. Currently, more than 30,000 structurally diverse secondary metabolites from marine sources have been isolated, making MNPs a profound, renewable source to investigate novel drug compounds. Marine sponges of the genus *Suberea* (family: Aplysinellidae) are recognized as producers of bromotyrosine derivatives, which are considered distinct chemotaxonomic markers for the marine sponges belonging to the order Verongida. This class of compounds exhibits structural diversity, ranging from simple monomeric molecules to more complex molecular scaffolds, displaying a myriad of biological and pharmacological potentialities. In this review, a comprehensive literature survey covering the period of 1998–2018, focusing on the chemistry and biological/pharmacological activities of marine natural products from marine sponges of the genus *Suberea*, with special attention to the biogenesis of the different skeletons of halogenated compounds, is presented.

## 1. Introduction

Oceans occupy almost 70% of the Earth’s surface, furnishing extraordinary biological and chemical diversity in different ecosystems on our planet [1]. Marine natural products (MNPs) have displayed a distinct track record as a rich and renewable source for novel drug leads [2]. Indeed, the majority of the newly discovered pharmacophores with potent biological/pharmacological activities are derived from marine sponges, corals, and tunicates, among other marine invertebrates [3]. Marine sponges (phylum: Porifera) are a large phylum within the animal Kingdom, and are considered prolific factories for producing bioactive natural products [4,5,6,7]. Currently, many MNPs and their derivatives are among the approved drugs on the market. These include anticancer drugs such as cytarabine (Cytosar-U®, DepoCyst®: FDA approval in 1969 for cancer), vidarabine (Vira-A®, approved by FDA in 1976 as an antiviral), trabectedin (Yondelis®, ET- 743, EU approval in 2011 for cancer), eribulin mesylate (Halaven®, FDA approval in 2010, and Heath Canada approval in 2011 for metastatic breast cancer), and brentuximab vidotin (Adcetris®, FDA approval in 2011 for Hodgkin’s lymphoma and in 2017 for cutaneous T-cell lymphoma), and other drugs such as ziconotide (Prialt®, approved by FDA in in 2004 as an analgesic for treatment of severe chronic pain) and ω-3 acid ethyl esters (Lovaza®, approved by FDA in 2004 for lowering blood triglyceride levels in adults with severe hypertriglyceridemia) [8,9,10]. Furthermore, more than twelve marine-derived compounds are currently under investigation in different clinical phases [2,8,9,10]. 

*Suberea* Bergquist, 1995 [11] is a genus of keratose (or horny) sponges that lacks a mineral skeleton, belonging to the Order Verongida (Family: Aplysinellidae). Species of *Suberea* have smooth or conulose surfaces and can be massive, stalked, or have branching growth forms. Their live coloration is usually bright and vivid (e.g., yellow, orange, brown, or red), with aerophobic pigments that darken when exposed to air. They have thick spongin fibers that form an irregular dendritic skeleton composed of both bark (external) and pith (central) fiber components, with the latter predominating. The bark component of the fibers is strongly laminated, which makes the fibers brittle. In addition, a dense, collagenous mesohyle between the fibers makes these sponges hard and barely compressible [11,12]. The World Porifera Database indicated that the genus *Suberea* currently contains 14 described species within *Suberea* Bergquist, 1995, including: *S. azteca* [13]; *S. clavata* [14]; *S. creba* [11]; *S. elegans* [15]; *S. etiennei* van Soest, Kaiser & Van Syoc, 2011 [16]; *S. flavolivescens* [17]; *S. fusca* [18]; *S. ianthelliformis* [15]; *S. laboutei* Bergquist [11]; *S. meandrina* [19]; *S. mollis* [20]; *S. pedunculata* [21]; *S. praetensa* [20]; and *S. purpureaflava* Gugel, Wagler & Brümmer, 2011 [22]. Sponges of the genus *Suberea* are found in shallow waters at depths from 8 to 55 meters, in both warm temperate and tropical waters off the coasts of Victoria, New South Wales, the Great Barrier Reef, Northwest Australia, Aru & Ki Islands, Indonesia, New Caledonia, Kermadec Islands, New Zealand, the Tropical Pacific Mexico, Clipperton Island in the Eastern Pacific, Caribbean Columbia, Gulf of Manaar, Sri Lanka, Southern India, Red Sea (Sudan and Egypt), Persian Gulf, Vema Seamount, and South Africa [23]. A common Indo-Pacific species, *S. ianthelliformis* (Lendenfeld, 1888), has a wide distribution over French Polynesia (Society, Marquesas, Tuamotu Archipelago Islands), Fiji, Solomon Islands, Northeast and Northwest Australia, the Philippines, South China Sea, Malaysia, and Indian Ocean Western Australia [24]. Like other marine sponge genera belonging to the order Verongida (Family: Aplysinellidae), such as *Aplysinella* and *Porphyria*, members of the genus *Suberea* are known to produce diverse structures of brominated tyrosine alkaloids [25,26] that display a myriad of bioactivities (Table 1) including cytotoxicity [27], antimicrobial properties [28], antibacterial properties [29], kinase inhibitor production [27], and antiproliferative properties [30]. Interestingly, a recent paper by Nicacio et al. [31] reported that a culture of the marine bacterium *Pseudovibrio denitrificans* Ab134, isolated from the Haplosclerida sponge *Arenosclera brasiliensis*, was able to produce bromotyrosine-derived alkaloids. This observation highlights important questions about the discretion in considering these brominated secondary metabolites as chemical markers of the order Verongida, and for marine sponge phylogeny in general. To the best of our knowledge, previous chemical investigations were mainly focused on only six species: *Suberea* sp., *S.* aff. *praetensa*, *S. mollis*, *S. creba*, *S. ianthelliformis*, and *S. clavata*. 

As a part of our ongoing research on biologically active marine natural products [32,33,34,35,36,37], this review comprehensively covers chemistry and biological activities of the isolated secondary metabolites from the marine sponges of the genus *Suberea,* reported over the period of 1998–2018, with a special attention to the halogenated compounds and their biosynthetic pathways. 

## 2. Chemistry and Biological Activities of Secondary Metabolites Isolated from the Members of the genus *suberea*

### 2.1. Halogenated Tyrosine Derivatives (Isoxazolines, Oxepinisoxazolines, and Phenolics)

**Figure 1:**
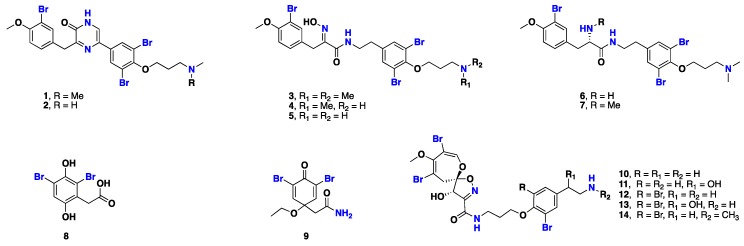
Chemical structures of **1**–**14.**

From Figure 1—seven cytotoxic bromotyrosine alkaloids, namely ma’edamines A and B (**1** and **2**) that possess a unique 2(*1H*) pyrazinone motif, along with aplysamine-2 (**3**), purpureamines H and I (**4** and **5**), and suberedamines A and B (**6** and **7**) were isolated from an Okinawan *Suberea* sp. [38]. Biosynthetically, **6** and **7**, which are precursors of **1** and **2**, could be formed by the condensation of two bromotyrosine units. Compounds **1** and **2** displayed in vitro cytotoxicity against murine leukemia L1210 and KB (mouth epidermoid carcinoma) cells, with IC_50_ values of 4.3, 3.9, 5.2 and 4.5 µg/mL, respectively. Furthermore, these compounds also exhibited inhibitory activity against c-erbB-2 kinase, with IC_50_ values of 6.7 and >10 µg/mL, respectively. Similarly, **6** and **7** exhibited in vitro cytotoxicity against murine leukemia L1201, with IC_50_ values of 8.0 and 8.6 μg/mL, and also against epidermoid carcinoma KB cells, with IC_50_ values of 9.0 and >10.0 μg/mL, respectively. Compounds **6** and **7** also showed antibacterial activity against *Micrococcus luteus*, with an MIC value of 12.6 µg/mL [27,38]. Furthermore, simple bromotyrosine derivatives isolated from *Suberea* sp., including subereaphenol K (**8**) and 2-(3,5-dibromo-1-ethoxy-4-oxocyclohexa-2,5-dien-1-yl) acetamide (**9**), showed cytotoxicity against NIH-3T3 (mouse embryonic fibroblast), HepG2 (human liver cancer), and HT-29 (human colon adenocarcinoma) cell lines [39]. Interestingly, psammaplysins I (**10**), J (**11**), A (**12**), B (**13**), and F (**14**), which all possess a complex spiro-oxepinisoxazoline scaffold, were also isolated from *Suberea* sp. [40] (Figure 1).

**Figure 2:**
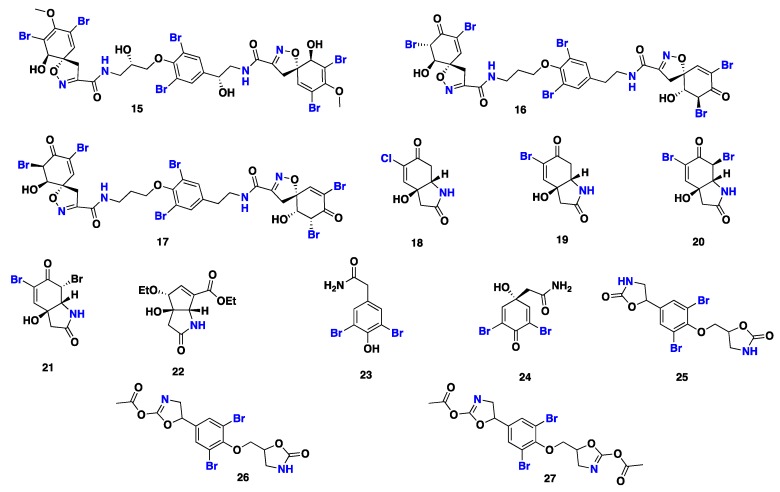
Chemical structures of **15**–**27.**

From Figure 2—three complex hexabromotyrosine derivatives containing the oxazolidone moiety, namely fistularin-3 (**15**) and 11,17-dideoxyagelorins A (**16**) and B (**17**), along with 5-chlorocavernicolin (**18**), 5-bromocavernicolin (**19**), cavernicolin-1 (**20**), cavernicolin-2 (**21**), subereatensin (**22**), 2-(3’,5’-dibromo-4’-hydroxyphenyl)acetamide (**23**), 3,5-dibromoverongiaquinol (**24**), and *bis*-oxazolidone (**25**) and its acetate congeners (**26** and **27**) were reported from the marine sponge *S*. aff. *praetensa* collected from the Gulf of Thailand [41,42,43]. Some of these compounds exhibited potent cytotoxicity against five human cancer cell lines: MCF-7 (breast cancer), NCI-H460 (human non-small cell lung cancer), SF268 (glioblastoma)*,* TK-10 (human renal carcinoma), and UACC-62 (human melanoma), with GI_50_ values in the micromolar range [41,42,43]. Moreover, **15,** which was also isolated from *Alisina archeri*, was shown to inhibit the growth of feline leukemia virus [44]. In addition to antitumor activity, **24** also displayed antibacterial activity [45]. Debitus et al. [46] found that **24,** isolated from *S. creba*, also exhibited a chloramphenicol antibiotic-like activity (quorum sensing inhibition) against the marine bacterium *Vibrio scala*. Weiss et al. [47] described the isolation of **24** from the marine sponge *Verongia aerophoba* and its antibacterial activity against eight different Gram-positive or Gram-negative marine bacteria, including *Alteromonas*, *Moraxella*, and *Vibrio* sp., in addition to potent activity against the marine bacterium *Photobacterium phosphoreum*, with an EC_50_ value of 3.45 µM. Moreover, this compound also inhibited the growth of the marine microalgae *Coscinodiscus wailesii* and *Prorocentrum minimum*, with an EC_50_ of 5.6 µM [47] (Figure 2). 

**Figure 3:**
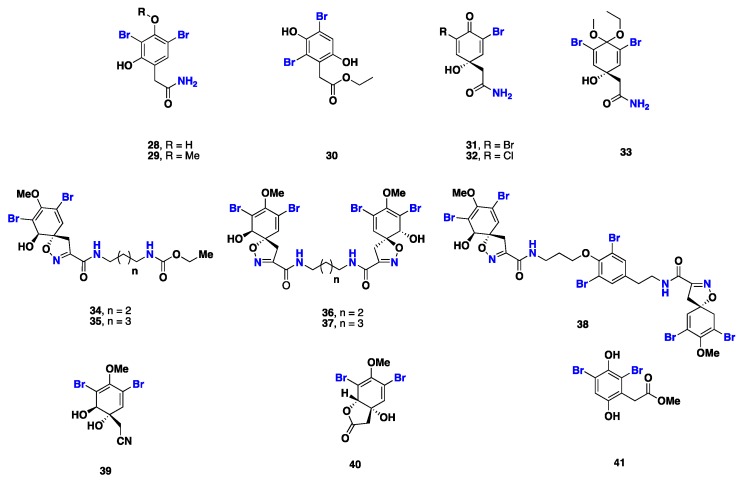
Chemical structures of **28**–**41.**

From Figure 3—six cytotoxic and antimicrobial dibromophenol derivatives, including subereaphenol A (**28**), 2-(3’,5’-dibromo-2’-hydroxy-4’-methoxyphenyl) acetamide (**29**), subereaphenol C (**30**), dibromoverongiaquinol (**31**), and bromochloroverongiaquinol (**32**), were isolated from *S. creba* [46], whereas **32** and 2-(3’,5’-dibromo-4’-ethoxy-1’-hydroxy-4’methoxy-2’,5’-cyclohexadien-1-yl) acetamide (**33**) were isolated from *S. mollis*, which was collected from the Egyptian Red Sea [48]. Compound **32** showed antibacterial activity against both Gram-positive (*Sarcina lutea*) and Gram-negative (*Alcaligena faecalis* and *Proteus vulgaris*) bacteria [49]. Compounds **23** and **30**–**32** [46] were re-isolated from *Suberea* sp., also collected from the Red Sea [50]. Compounds **23**, **30** and **32** exhibited cytotoxic and antiproliferative effects against HCT-116 (human colon cancer) and HeLa (human carcinoma) cell lines, and **32** was found to be the most cytotoxic, with IC_50_ values of 4.5 and 10 µg/mL, respectively. Additionally, **32** also exhibited moderate antibacterial activity against *Escherichia coli*, with an inhibition zone of 12 mm [50]. 

The oxazolidone-containing metabolites subereamollines A (**34**) and B (**35**), aerothionin (**36**), homoaerothionin (**37**), 11,19-dideoxyfistularin-3 (**38**), and (+)-aeroplysinin-1 (**39**) were reported from *S. creba* [46]. While **36** displayed a feed chemical defense role against the predatory fish *Blennius sphinx* [51], **39** displayed potent antibacterial activity against *Staphylococcus albus*, *Bacillus cereus*, and *B. subtilis*, with MIC values of 20–100 µg/mL [52,53], as well as cytotoxicity against a panel of tumor cell lines, including human cervix uteri, Ehrlich ascites tumor (EAT), and HeLa cell lines [54,55,56,57]. Moreover, synthetic congeners of (+)-aeroplysinin-1 showed an in vivo inhibition of the receptor tyrosine kinase (RTK) and antiproliferative activity [58]. Aeroplysinin-2 (**40**) and subereaphenol B (**41**), previously reported from the Red Sea *Suberea* sp. [30], were re-isolated from *S. mollis* also collected from the Red Sea [59] (Figure 3).

**Figure 4:**
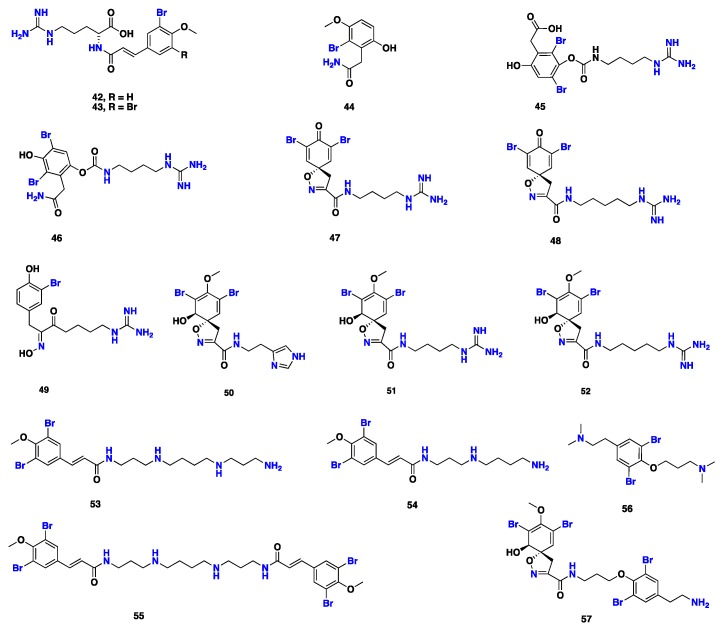
Chemical structures of **42**–**57**.

From Figure 4—two antimicrobial brominated arginine derivatives, subereamines A and B (**42** and **43**), along with subereaphenol D (**44**), were isolated from *S. mollis* collected in the Egyptian Red Sea [28]. The chemical investigation of *S. clavata* extracts furnished eight guanidine-containing bromotyrosine derivatives, namely clavatadines A–E (**45**–**49**), aerophobin-1 (**50**), purealidin L (**51**), and aplysinamisine II (**52**) that showed inhibition against Factor XIa [60,61]. The antibacterial bromotyrosine alkaloids possessing polyamine motifs, ianthelliformisamines A–C (**53**–**55**), were isolated from *S. ianthelliformis* along with aplysamine-1 (**56**) and araplysillin-I (**57**). Compound **53** showed antibacterial activity against the Gram-negative bacterium *Pseudomonas aeruginosa*, with an IC_50_ of 6.8 μM [29] (Figure 4). 

**Figure 5:**
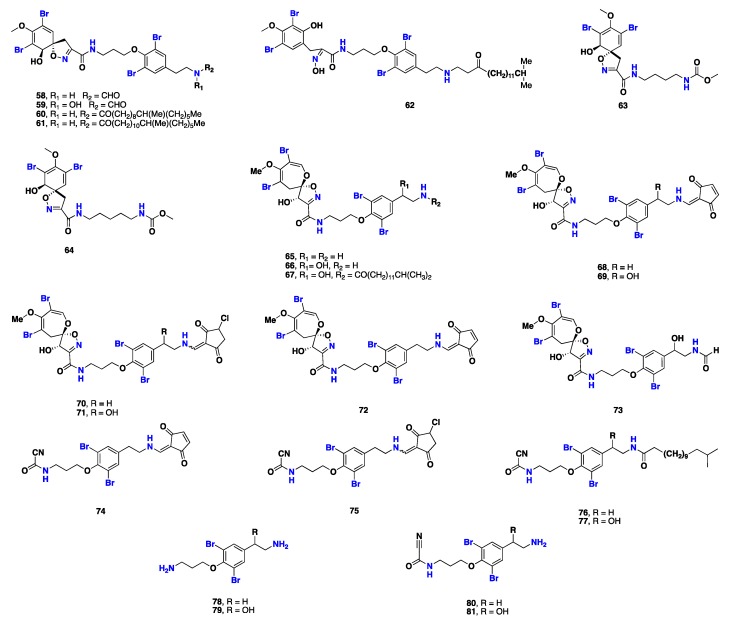
Chemical structures of **58**–**81**.

From Figure 5—five antiplasmodial metabolites, including araplysillin *N*-20-formamide (**58**), araplysillin *N*-20-hydroxyformamide (**59**), araplysillin IV (**60**), araplysillin V (**61**), and araplysillin VI (**62**), were reported from *S. ianthelliformis*. Compounds **58**–**62** exhibited weak to moderate inhibitory activities against both chloroquine-resistant and chloroquine-sensitive *Plasmodium falciparum* strains FcB-1 and 3D7, with IC_50_ values in the range of 1.0 to 59 µM, and 0.9 to 19.9 µM, respectively [62]. Subereamollines C and D (**63** and **64**), isolated from *Suberea* sp. collected from the Red Sea, displayed weak antiproliferative activity [30]. Psammaplysins A (**65**), B (**66)**, D (**67**), E (**68**), 19-hydoxypsammaplysin E (**69**), psammaplysin X (**70**), 19-hydroxypsammaplysin X (**71**), psammaplysin Y (**72**), 19-hydroxyceratinamide A (**73**), subereamides A–C (**74**–**76**), and 12-hydroxysubereamide C (**77**), in addition to moloka’iamine (**78**), hyroxymoloka’iamine (**79**), ceratinamine (**80**) and hydroxyceratinamine (**81**), were isolated from a Micronesian sponge *Suberea* sp. These psammaplysin analogues (**65**–**81**) displayed potent cytotoxicity against six human tumor cell lines, namely HCT-15 (colon cancer), PC-3 (prostate cancer), ACHN (renal cancer), MDA-MB-231 (breast cancer), NUGC-3 (stomach cancer), and NCI-H23 (lung cancer), with GI_50_ values as low as 0.8 μM. This suggests these compounds could serve as promising molecular templates for the development of anticancer agents [63] (Figure 5).

**Figure 6:**
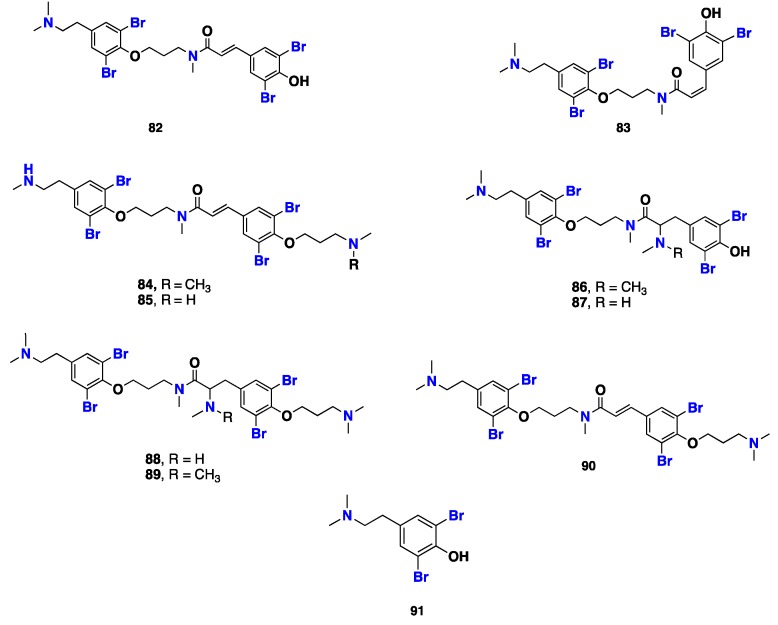
Chemical structures of **82**–**91**.

From Figure 6—Al-Mourabit et al. [64] reported the isolation of eight tetrabromotyrosine alkaloids, psammaplysenes F–I (**82**–**85**), and anomoians C–F (**86**–**89**), along with the known natural products psammaplysene D (**90**) and *N,N*-dimethyldibromotyramine (**91**), from the Polynesian sponge *S. ianthelliformis*. Compounds **82**, **83** and **86**–**89** exhibited moderate cytotoxicity against the KB cell line, whereas **90** was the most potent with an IC_50_ of 0.7 µM. Although the structures of **88** and **89** resemble that of **90**, they exhibited weaker cytotoxicity than **90**. It can be hypothesized that the presence of the double bond in the 3,5-dibromo *p*-hydroxycinnamoyl moiety in **90**, instead of the amino or alkylamino group on the carbon adjacent to the amide carbonyl in **88** and **89**, was essential for this activity. Curiously, **82**, which contains a double bond in the 3,5-dibromo *p*-hydroxycinnamoyl moiety as in **90,** but lacks the *N,N*-dimethylaminopropyl substituent on the phenolic hydroxyl group of the 3,5-dibromo *p*-hydroxycinnamoyl moiety, displayed much weaker cytotoxicity than **90**. Therefore, both *N,N*-dimethylaminopropyl and *trans*-3,5-dibromo *p*-hydroxycinnamoyl moieties seem to be essential for the cytotoxicity for this series of compounds. Moreover, **90** showed a promising in vitro acetylcholinesterase inhibitory activity with an IC_50_ of 1.3 µM, as well as a potent activity against fish antifeedant activity [34,64] (Figure 6).

### 2.2. Non-halogenated Derivatives (Tyrosine, Aaptamine, Pyrrole, Quinolines, Isopernoids, Sesterterpenoids and Macrolides)

**Figure 7:**
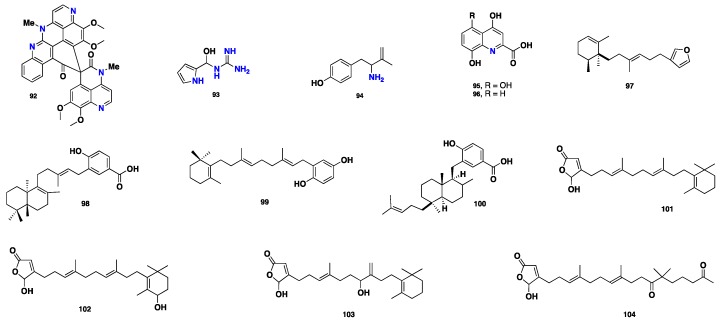
Chemical structures of **92**–**104**.

From Figure 7—lihouidine (**92**), a polycyclic alkaloid featuring two modified aaptamine moieties, was obtained from *Suberea* n. sp. collected in Australia. Compound **92** displayed moderate cytotoxicity against P388D1 (mouse lymphoma cells) with an IC_50_ of 3 µg/mL [65]. Interestingly, structurally less complex metabolites such as 1-(hydroxy(1*H*-pyrrol-2-yl)methyl)guanidine (**93**) and 4-(2-amino-3-methylbut-3-en-1-yl) phenol (**94**) were also isolated from the Red Sea *Suberea* sp. Compound **93** exhibited low cytotoxicity against HCT-116 and HeLa cell lines, with IC_50_ values of 25 and 30 µg/mL, respectively, whereas **94** showed moderate cytotoxicity with IC_50_ values of 20 and 27 µg/mL, respectively. Furthermore, **93** and **94** displayed moderate antifungal activity against *Candida albicans* at a concentration of 100 µg, with inhibition zones of 8 and 15 mm, respectively [50]. 5-Hydroxyxanthenuric acid (**95**) and xanthurenic acid (**96**) were co-isolated, along with **82–91,** from the Polynesian *S. ianthelliformis* [64]. 

A few non-brominated metabolites, including terpenoid compounds such as (+)-(5*S*,6*S*)-subersin (**97**) and three meroditerpenoids including (−)-subersic acid (**98**), jaspaquinol (**99**), and (-)-jaspic acid (**100**), were also reported from *Suberea* sp. These compounds showed inhibitory activity against human 15-lipoxygenase, with IC_50_ values >100, 15, 0.3, and 1.4 µM, respectively [66]. Four sesterterpenoids, namely luffariellolide (**101**), 18-hydroxyluffariellolide (**102**), acantholides A (**103**), and C (**104**), were reported from *Suberea* sp. collected from the Philippines [67]. These naturally occurring compounds, along with synthetically prepared analogues, were evaluated for their antimicrobial activity against two Gram-negative bacterial strains, *Klebsiella *pneumoniae** and *Salmonella enterica*. Compound **101** displayed moderate activity against *S. enterica* with an MIC value of 4 µg/mL, but no activity against *K. pneumoniae* (MIC value >64 µg/mL), while **102** exhibited moderate activity against *K. pneumoniae* (MIC value of 8 µg/mL) and weak activity against *S. enterica* (MIC value 16 µg/mL) [67] (Figure 7).

**Figure 8:**
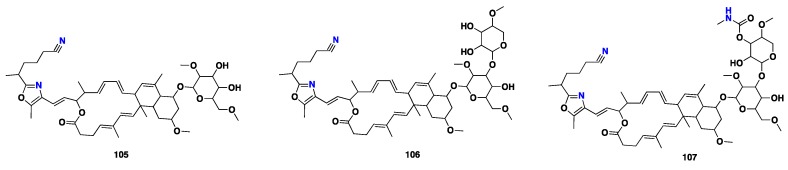
Chemical structures of **105–107**.

From Figure 8—additionally, three potent cytotoxic glycosylated oxazole-bearing macrolides, **105**–**107,** were isolated from *S. creba* collected in New Caledonia. These compounds exhibited strong cytotoxicity against seven tumor cell lines, including A549 (human lung carcinoma), BxPC3 (Human primary pancreatic adenocarcinoma), KB, KB-V1 (human cervix carcinoma), LoVo (human colon carcinoma), Namalwa (human Burkitt lymphoma), and SKOV3 ovarian carcinoma), with EC_50_ values ranging from micromolar to picomolar [68,69] (Figure 8). 

**Figure 9:**
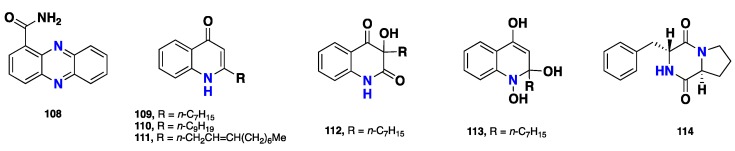
Chemical structures of **108**–**114**.

From Figure 9—curiously, only few metabolites have been reported from *Suberea* sponge-associated microorganisms. These include a dibenzopyrazine alkaloid (**108**) and five quinolone derivatives (**109–113**), along with the 2,5-diketopeiprazine alkaloid (**114)** produced by a marine bacterium *Pseudomonas* sp. isolated from *S. creba*. Compound **109** displayed promising in vitro antibacterial activity against the marine bacterium *V. scala* [46] (Figure 9).

**Table 1:** Summary of secondary metabolites isolated from marine sponges of the genus *Suberea,* and their biological activities.

Compound	Species	Local of Collection	Biological Activity	References
**1–5**	*Suberea* sp.	Okinawa	Cytotoxic, kinase inhibitors	[27]
**6–7**	*Suberea* sp.	Okinawa	Cytotoxic, antibacterial	[38]
8–9	*Suberea* sp.	Okinawa	Cytotoxic	[39]
**10–14**	*Suberea* sp.	Guam	Nr	[40]
**15–27** **24**	*S.* aff. *praetensa* *S. creba*	Thailand Coral Sea, Australia	Cytotoxic Antiviral, antibacterial	[41,42,43] [45,46,47]
**28–32** **32–33** **23, 30–32**	*S. creba* *S. mollis* *Suberea* sp.	Coral Sea, Australia Red Sea, Egypt Red Sea, Egypt	Cytotoxic, antimicrobial Cytotoxic, antimicrobial Cytotoxic, antiproliferative, antibacterial	[46] [48,49] [50]
**34–39**	*S. creba*	Coral Sea, Australia	Antimicrobial, Cytotoxic, tyrosine kinase inhibitor, antiproliferative	[46,51,52,53] [54,55,56,57,58]
**40–41**	*Suberea* sp. *S. mollis*	Red sea, Egypt Red Sea, Egypt	Cytotoxic, antioxidant Nr	[30] [59]
**42–44**	*S. mollis*	Red Sea, Egypt	Antimicrobial	[28]
**45–52**	*S. clavata*	Great Barrier Reef, Australia	Plasma thromboplastin inhibitor	[60,61]
**53–57**	*S. ianthelliformis*	Manta Ray Bommie, Australia	Antibacterial	[29]
**58–62**	*S. ianthelliformis*	Solomon Islands	Antiplasmodial	[62]
**63–64**	*Suberea* sp.	Red Sea, Egypt	Antiproliferative	[30]
**65–81**	*Suberea* sp.	Micronesia	Cytotoxic	[63]
**82–91**	*S. ianthelliformis*	French Polynesia	Cytotoxic, acetylcholinesterase inhibitor	[34,64]
**92**	*Suberea* sp.	Lihou Reef, Australia	Cytotoxic	[65]
**93–94**	*Suberea* sp.	Red Sea, Egypt	Cytotoxic, antimicrobial	[50]
**95–96**	*S. ianthelliformis*	French Polynesia	Nr	[64]
**97–100**	*Suberea* sp.	Papua New Guinea	Human 15-Lipoxygenase inhibitor	[66]
**101–104**	*Suberea* sp.	Philippines	Antimicrobial	[67]
**105–107**	*S. creba*	New Caledonia	Cytotoxic	[68,69]
**108–114**	*S. creba*	New Caledonia	Antibacterial	[46]

Nr: Not reported.

## 3. Proposed Biogenetic Pathways for Different Bromotyrosine Derivatives

**Figure 10:**
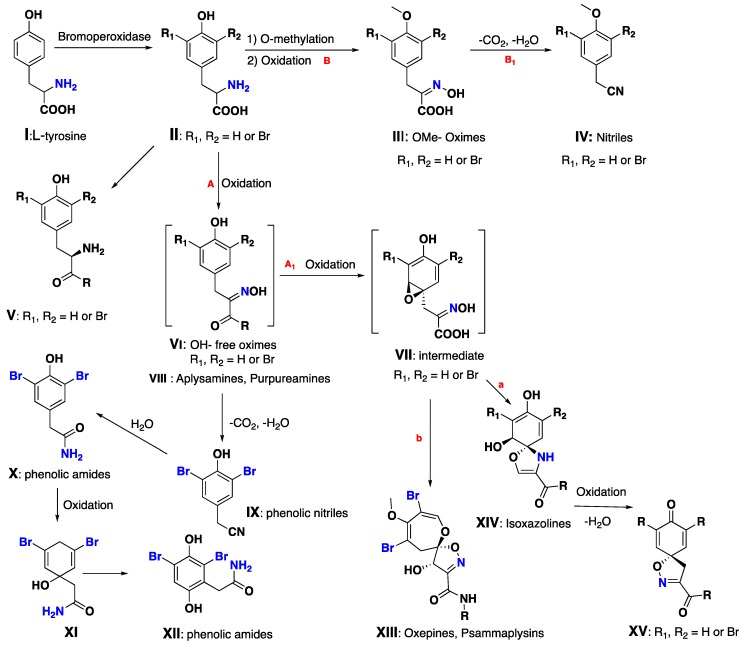
Proposed biogenetic pathway of different bromotyrosine derivatives.

From Figure 10—earlier biosynthetic studies on bromotyrosine derivatives showed that the metabolic cascade is initiated by bromination of tyrosine (**I**) with bromoperoxidase enzymes to give a brominated tyrosine intermediate **II**. Then, **II** can be transformed into **V** (like purpurealidins A–F) or can undergo further reactions. Route **A**: Oxidation of the amine to an oxime, affording the intermediate **VI**. Route **B**: *O*-methylation of **II**, followed by the oxidation of the amine functionality to an oxime (**III**) or producing compounds such as purpuramines and aplysamines (**VIII**). Moreover, the first pathway (route **A**) could also afford phenolic nitriles (**IX**) and amides (**XII**), or alternatively, can undergo an epoxidation (**A_1_**) to form an intermediate **VII**. This in turn leads to either (**a**) the isoxazoline ring system (**XIV**), producing metabolites such as aerothionin, homoaerothionin, purealdin Q, and purpurealidins A and J, or (**b**) the oxepine ring system (**XIII**), as found in the psammaplysins. Moreover, the isoxazoline ring (**XIV**) can undergo further oxidation and dehydration leading to **XV** (like purpurealidin B). On the other hand, route **B** could lead to the pathway featuring a dehydration/decarboxylation (**B_1_**) to afford the *O*-methylated nitriles (**IV**) [26,70,71,72] (Figure 10).

## 4. Conclusions

The present review highlights a comprehensive literature survey covering the chemical and biological remarks of secondary metabolites isolated from marine sponges belonging to the genus *Suberea* over the period of 1998–2018. One hundred and fourteen isolated metabolites are categorized into two main groups, presenting an array of molecular architectures that display a vast spectrum of bioactivities. Additionally, a brief insight of the proposed biogenetic pathways leading to different bromotyrosine motifs is also discussed. The chemodiversity and bioactivities of the metabolites from the sponges of this genus make them interesting targets for further exploration to obtain novel compounds with therapeutic potentiality. Furthermore, this systematic review provides evidence that a myriad of secondary metabolites reported from members of the genus *Suberea* are structurally unique and exhibit a variety of biological/pharmacological activities, although cytotoxic activity predominated. Moreover, it can be observed that the habitats of these sponges also influence the types of compounds and consequent biological activities. These findings can be helpful in the bioprospecting process of marine sponges of this genus, and in finding new compounds as potential targets for further drug development in different therapeutic areas. 

## Abbreviations

The following abbreviations are used in this manuscript:

GI_50_	Half maximal growth inhibition
Factor XIa	Plasma thromboplastin antecedent
IC_50_	Half maximal inhibitory concentration
MIC	Minimum Inhibitory concentration

## References

[1] Bourguet-Kondracki M.L., Kornprobst J.M., La Barre S., Kornprobst J.M. (2014). Promising Marine Molecules in Pharmacology. Outstanding Marine Molecules.

[2] Jiménez C. (2018). Marin natural products in medicinal chemistry. ACS Med. Chem. Lett..

[3] Mehbub M.F., Lei J., Franco C., Zhang W. (2014). Marine Sponge Derived Natural Products between 2001 and 2010: Trends and Opportunities for Discovery of Bioactives. Mar. Drugs.

[4] Martins A., Vieira H., Gaspar H., Santos S. (2014). Marketed marine natural products in the pharmaceutical and cosmeceutical Industries: Tips for success. Mar. Drugs.

[5] Blunt J.W., Copp B.R., Keyzers R.A., Munro M.H., Prinsep M.R. (2016). Marine natural products. Nat. Prod. Rep..

[6] Blunt J.W., Copp B.R., Keyzers R.A., Munro M.H., Prinsep M.R. (2017). Marine natural products. Nat. Prod. Rep..

[7] Blunt J.W., Carroll A.R., Copp A.R., Davis R.A., Keyzers R.A., Prinsep M.R. (2018). Marine natural Products. Nat. Prod. Rep..

[8] Montaser R., Luesch H. (2011). Marine natural products: A new wave of drugs. Future Med. Chem..

[9] Rangel M., Falkenberg M. (2015). An overview of the marine natural products in clinical trials and on the market. J. Coast. Life Med..

[10] Patridge E., Gareiss P., Kinch M.S., Hoyer D. (2016). An analysis of FDA-approved drugs: Natural products and their derivatives. Drug Discov. Today.

[11] Bergquist P.R. (1995). Dictyoceratida, Dendroceratida and Verongida from the New Caledonia Lagoon (Porifera: Demospongiae). Mem. Queensl. Mus..

[12] Hooper J.N.A., Van Soest R.W.M. (2002). Systema Porifera. A Guide to the Classification of Sponges.

[13] Gómez P., Bakus G. (1992). Aplysina gerardogreeni and Aplysina aztecus (Porifera: Demospongiae) New Species from the Mexican Pacific.

[14] Pulitzer-Finali G. (1982). Some new or little-known sponges from the Great Barrier Reef of Australia. Bol. Mus. Istituti Biol. Univ. Genova.

[15] Lendenfeld R. (1888). Descriptive Catalogue of the Sponges in the Australian Museum, Sidney.

[16] Van Soest R.W.M., Kaiser K., van Syoc R. (2011). Sponges from Clipperton Island, East Pacific. Zootaxa.

[17] Hofman C.C., Kielman M. (1992). The excavating sponges of the Santa-Marta area, Colombia, with description of a new species. Bijdr. Dierkd..

[18] Carter H.J. (1880). Report on specimens dredged up from the Gulf of Manaar and presented to the Liverpool Free Museum by Capt. W. H. Cawne Warren. Ann. Mag. Nat. Hist..

[19] Kelly M., Amirapu S., Mills S., Page M., Reiswig H.M. (2015). Kermadec Islands sponge biodiversity: A review and description of a new species, *Suberea meandrina* sp. nov. (Demospongiae, Verongiida, Aplysinellidae). Bull. Auckland Mus..

[20] Row R.W.H. (1911). Reports on the marine biology of the Sudanese Red Sea, from collections made by Cyril Crossland, M.A., B.Sc., F.Z.S. XIX. Report on the sponges collected by Mr. Cyril Crossland in 1904-5. Part II. Non-calcarea. J. Linn. Soc. Zool..

[21] Lévi C. (1969). Spongiaires du Vema Seamount (Atlantique Sud). Bull. Mus. Natl. Hist. Nat..

[22] Gugel J., Wagler M., Brümmer F. (2011). Porifera, one new species *Suberea purpureaflava* n. sp. (Demospongiae, Verongida, Aplysinellidae) from northern Red Sea coral reefs, with short descriptions of Red Sea Verongida and known *Suberea* species. Zootaxa.

[23] Van Soest R.W.M., Boury-Esnault N., Hooper J.N.A., Rützler K., de Voogd N.J., Alvarez B., Hajdu E., Pisera A.B., Manconi R., Schönberg C. (2018). World Porifera Database. http://www.marinespecies.org/porifera.

[24] Hall K.A., Hooper J.N.A. (2014). QM0012 *Suberea ianthelliformis* (Lendenfeld, 1888). SpongeMaps: An Online Community for Taxonomy and Identification of Sponges. http://www.spongemaps.org.

[25] Abou El-Ezz R., Ibrahim A., Habib E., Wahba A., Kamel H., Afifi M., Hassanean H., Ahmed S. (2017). Review of natural products from marine organisms in the Red Sea. Int. J. Pharm. Sci. Res..

[26] Peng J., Li J., Hamann M.T., Cordell G.A. (2005). The Marine Bromotyrosine Derivatives. The Alkaloids: Chemistry and Biology.

[27] Hirano K., Kubota T., Tsuda M., Watanabe K., Fromont J., Kobayashi J. (2002). Ma’edamines A and B, cytotoxic bromotyrosine alkaloids with a unique 2 (1H) pyrazinone ring from Sponge *Suberea* sp.. Tetrahedron.

[28] Shaala L.A., Bamane F.H., Badr J.M., Youssef D.T.A. (2001). Brominated arginine-derived alkaloids from the Red Sea sponge *Suberea mollis*. J. Nat. Prod..

[29] Xu M., Davis R.A., Feng Y., Sykes M.L., Shelper T., Avery V.M., Camp D., Quinn R.J. (2012). Ianthelliformisamines A-C, antibacterial bromotyrosine-derived metabolites from the marine sponge *Suberea ianthelliformis*. J. Nat. Prod..

[30] Shaala L.A., Youssef D.T.A., Badr J.M., Sulaiman M., Kherd A. (2015). Bioactive brominated metabolites from the Red Sea sponge *Suberea mollis*. Mar. Drugs.

[31] Nicacio K.J., Ióca L.P., Fróes A.M., Leomil L., Appolinario L.R., Thompson C.C., Thompson F.L., Ferreira A.G., Williams D.E., Andersen R.J. (2017). Cultures of the marine bacterium *Pseudovibrio denitrificans* Ab134 produce bromotyrosine-derived alkaloids previously only isolated from marine sponges. J. Nat. Prod..

[32] El-Demerdash A., Moriou C., Martin M.T., Rodrigues-Stien A., Petek S., Demoy-Schnider M., Hall K., Hooper J.N.A., Debitus C., Al-Mourabit A. (2016). Cytotoxic guanidine alkaloids from a French Polynesian *Monanchora* n. sp. sponge. J. Nat. Prod..

[33] El-Demerdash A., Moriou C., Martin M.T., Petek S., Debitus C., Al-Mourabit A. (2017). Unguiculins A-C: Cytotoxic *bis*-guanidine alkaloids from the French Polynesian sponge, *Monanchora* n. sp.. Nat. Prod. Res..

[34] El-Demerdash A. (2016). Isolation of Bioactive Marine Natural Products and Bio-Inspired Synthesis of Fused Guanidinic Tricyclic Analogues. Ph.D. Thesis.

[35] El-Demerdash A., Atanasov A.G., Bishayee A., Abdel-Mogib M., Hooper J.N.A., Al-Mourabit A. (2018). *Batzella*, *Crambe* and *Monanchora*: Highly prolific marine sponge genera yielding compounds with potential applications for cancer and other therapeutic areas. Nutrients.

[36] El-Demerdash A., Tammam M.A., Atanasov A.G., Hooper J.N.A., Al-Mourabit A., Kijjoa A. (2018). Chemistry and biological activities of the marine sponges of the genera *Mycale* (*Arenochalina*), *Biemna* and *Clathria*. Mar. Drugs.

[37] El-Demerdash A., Petek S., Debitus C., Al-Mourabit A. (2018). Fatty Acids Pattern from the French Polynesian *Monanchora* n. sp. Marine Sponge. Chem. Nat. Compd..

[38] Tsuda M., Sakuma Y., Kobayashi J. (2001). Suberedamines A and B, new bromotyrosine alkaloids from a sponge *Suberea* species. J. Nat. Prod..

[39] Shaker K.H., Zinecker H., Ghani M.A., Imhoff J.F., Schneider B. (2010). Bioactive metabolites from the sponge *Suberea* sp.. Chem. Biodivers..

[40] Wright A.D., Scupp P.J., Scror J.P., Engemann A., Rohde S., Kelmna D., Voogd N., Carroll A., Motti C.A. (2012). Twilight zone sponges from Guam yield theonellin isocyanate and psammaplysins I and J. J. Nat. Prod..

[41] Kijjoa A., Watanadilok R., Sonchaeng P., Silva A.M.S., Eaton G., Herz W. (2001). 11,17-Dideoxyagelorin A and B, new bromotyrosine derivatives and analogs from the marine sponge *Suberea* aff. *praetensa*. Z. Naturforsch..

[42] Kijjoa A., Watanadilok R., Sonchaeng P., Sawangwong P., Pedro M., Nascimento M.S.J., Silva A.M.S., Eaton G., Herz W. (2002). Further halotyrosine derivatives from the marine sponge *Suberea* aff. *praetensa*. Z. Naturforsch..

[43] Kijjoa A., Watanadilok R., Sonchaeng P., Puchakarn S., Sawangwong P., Herz W. (2004). Bromotyrosine derivatives from the marine sponge *Suberea* aff. *praetensa*. Bol. Mus. Ist. Biol. Univ. Genova.

[44] Gunasekera S.P., Cross S.S. (1992). Fistularin-3 and 11-ketofistularin-3. Feline leukemia virus active bromotyrosine metabolites from the marine sponge *Aplysina archeri*. J. Nat. Prod..

[45] Sharma G.M., Burkholder P.R. (1967). Studies on the antimicrobial substances of sponges II. Structure and synthesis of a bromine-containing antibacterial, compound from a marine sponge. Tetrahedron Lett..

[46] Debitus C., Guella G., Mancini I., Waikedre J., Guemas J.P., Nicolas J.L., Pietra F. (1998). Quinolones from a bacterium and tyrosine metabolites from its host sponge, *Suberea creba* from the Coral Sea. J. Mar. Biotechnol..

[47] Weiss B., Ebel R., Elbrächter M., Kirchner M., Proksch P. (1996). Defense metabolites from the marine sponge *Verongia aerophoba*. Biochem. Syst. Ecol..

[48] Shaala L.A., Khalifa S.I., Mesbah M.K., van Soest R.W.M., Youssef D.T.A. (2008). Subereaphenol A, a new cytotoxic and antimicrobial dibrominated phenol from the Red Sea sponge *Suberea mollis*. Nat. Prod. Commun..

[49] D’Ambrosio M., Gueriero A., Pietra F. (1984). Novel, racemic or nearly-racemic antibacterial bromo- and chloroquinols and γ-lactams of the verongiaquinol and the cavernicolin type from the marine sponge *Aplysina* (=*Verongia*) *cavernicola*. HeIv. Chim. Acta.

[50] Shaala L.A., Almohammadi A. (2017). Biologically active compounds form the Red Sea sponge *Suberea* sp.. Pak. J. Pharm. Sci..

[51] Thomas C., Wolff W., Padmakumar K., Ebel R., Proksch P.Z. (2004). Chemical defense of Mediterranean sponges *Aplysina cavernicola* and *Aplysina aerophoba*. Z. Naturforsch..

[52] Fattorusso E., Minale L., Sodano G. (1972). Aeroplysinin-1, an antibacterial bromo-compound from the sponge *Verongia aerophoba*. Chem. Soc. Perkin Trans..

[53] Fulmor W., Van Lear G.E., Morton G.O., Mills R.D. (1970). Isolation and absolute configuration of the aeroplysinin I enantiomorphic pair from *Ianthella ardis*. Tetrahedron Lett..

[54] Teeyapant R., Woerdenbag H.J., Kreis P., Hacker J., Wray V., Witte L., Proksch P. (1993). Antibiotic and cytotoxic activity of brominated compounds from the marine sponge *Verongia aerophoba*. Z. Naturforsch..

[55] Koulman A., Proksch P., Ebel R., Beekman A.C., van Uden W., Konings A.W., Pedersen J.A., Pras N., Woerdenbag H.J. (1996). Cytoxicity and mode of action of aeroplysinin-1 and a related dienone from the sponge *Aplysina aerophoba*. J. Nat. Prod..

[56] Kreuter M.H., Leake R.E., Rinaldi F., Müller-Klieser W., Maidhof A., Müller W.E.G., Schröder H.C. (1990). Inhibition of intrinsic protein tyrosine kinase activity of EGF-receptor kinase complex from human breast cancer cells by the marine sponge metabolite (+)-aeroplysinin-1. Comp. Biochem. Physiol. B Biochem. Mol. Biol..

[57] Martinez-Poveda B., Garcia-Vilas J.A., Cardenas C., Melgarejo E., Quesada A.R., Medina M.A. (2013). The brominated compound aeroplysinin-1 inhibits proliferation and the expression of key pro-inflammatory molecules in human endothelial and monocyte cells. PLoS ONE.

[58] Hinterdinga K., Knebelb A., Herrlichb P., Waldmanna H. (1998). Synthesis and biological evaluation of aeroplysinin analogues: A new class of receptor tyrosine kinase inhibitors. Bioorg. Med. Chem..

[59] Abou-Ashour M.I., Shaala L.A., Youssef D.T.A., Bader J.M., Habib A.M. (2008). Bioactive brominated metabolites from the Red Sea sponge *Suberea mollis*. J. Nat. Prod..

[60] Buchanan M.S., Carroll A.R., Wessling D., Jobling M., Avery V.M., Davis R.A., Feng Y., Xue Y., Oster L., Fex T. (2008). Clavatadine A, a natural product with selective recognition and irreversible inhibition of factor Xia. J. Med. Chem..

[61] Buchanan M.S., Carroll A.R., Wessling D., Jobling M., Avery V.M., Davis R.A., Feng Y., Hooper J.N.A., Quinn R.J. (2009). Clavatadines C-E, guanidine alkaloids from the Australian sponge *Suberea clavata*. J. Nat. Prod..

[62] Mani L., Jullian V., Mourkazel B., Valentin A., Dubois J., Cresteil T., Folcher E., Hooper J.N.A., Erpenbeck D., Aalbersberg W. (2012). New antiplasmodial bromotyrosine derivatives from *Suberea ianthelliformis* (Lendenfeld, 1888). Chem. Biodivers..

[63] Lee Y.J., Han S., Lee H.S., Kang J.S., Yun J., Sim C.J., Shin H.J., Le J.S. (2013). Cytotoxic psammaplysin analogues from a *Suberea* sp. marine sponge and the role of the spirooxepinisoxazoline in their activity. J. Nat. Prod..

[64] El-Demerdash A., Moriou C., Toullec J., Besson M., Soulet S., Schmitt N., Petek S., Lecchini D., Debitus C., Al-Mourabit A. (2018). Bioactive bromotyrosine-derived alkaloids from the Polynesian sponge *Suberea ianthelliformis*. Mar. Drugs.

[65] Bowden B.F., McCool B.J., Willis R.H. (2004). Lihouidine, a novel spiro polycyclic aromatic alkaloids from the marine sponge *Suberea* n. sp. (Aplysinellidae, Verongida). J. Org. Chem..

[66] Carroll J., Jonsson E.N., Ebel R., Hartman M.S., Holman T.R., Crews P. (2001). Probing sponge-derived terpenoids for human 15-lipoxgenase inhibitors. J. Org. Chem..

[67] Lee J., Shin A.Y., Lee H.S. (2017). Isolation and synthesis of luffariellolide derivatives and evaluation of antibacterial activities against Gram-Negative bacteria. Bull. Korean Chem. Soc..

[68] Carletti I., Massiot G. (2015). Macrolides Useful as Anticancer Agents. U.S. Patent.

[69] Carletti I., Massiot G. (2018). Macrolides Useful as Anticancer Agents. U.S. Patent.

[70] Shimizu Y. (1984). Paralytic shellfish poisons. Prog. Chem. Org. Nat. Prod..

[71] Butler A., Walker J.V. (1993). Marine haloperoxidases. Chem. Rev..

[72] Mani L. (2005). The isolation and characterization of antibacterial compounds from the marine sponge, *Suberea clavata*. Master’s Thesis.

